# Psychometric Validation of the Portuguese Version of the Modern Homonegativity Scale among Portuguese College Students

**DOI:** 10.3390/ejihpe12080081

**Published:** 2022-08-18

**Authors:** Ana Belén García-Berbén, Henrique Pereira, Adrián S. Lara-Garrido, Gloria Álvarez-Bernardo, Graça Esgalhado

**Affiliations:** 1 Campus Universitario de Cartuja, University of Granada, Calle Prof. Vicente Callao, 3, 18011 Granada, Spain; 2 Department of Psychology and Education, Faculty of Social and Human Sciences, University of Beira Interior, Pólo IV, 6200-209 Covilhã, Portugal; 3 Research Centre in Sports Sciences, Health Sciences and Human Development (CIDESD), 5001-801 Vila Real, Portugal

**Keywords:** modern homonegativity, gay men, lesbian woman, college students, homophobia, attitudes, modern prejudice, psychometry

## Abstract

The studies focused on analysing attitudes toward homosexuality show that the manifestation of homonegativity has evolved into more modern forms. We therefore propose using instruments that capture subtle aspects in discrimination against gay and lesbian people. The objective of this study is twofold. First, we aim to adapt and validate the Portuguese version of the Modern Homonegativity Scale. Second, we set out to analyse the modern homonegativity shown by Portuguese university students. The scale includes two parallel subscales (MHS-Gay Men and MHS-Lesbian Women), each with 12 items. Six hundred and forty-one Portuguese college students between 18 and 27 years of age participated in the study (M_age_ = 21.23; SD = 1.88). The results demonstrate the unidimensionality of the scale and a high degree of internal consistency, along with satisfactory fit indices. Those people who identified as male and heterosexual showed greater modern homonegativity. We conclude that the Portuguese version of the MHS is valid and reliable for evaluating modern homonegativity in Portugal.

## 1. Introduction

The treatment of people depends on the characteristics that each society takes as acceptable. In Europe, there are differences in the acceptance given to gay, lesbian and bisexual people. The study carried out by D’Amore et al. [1] compares seven European countries, and places Portugal among those that have the most progressive legislation regarding LGTB matters. This study concludes that the Portuguese population shows a higher level of support of equal marriage rights and same-sex families, as opposed to more politically conservative countries such as Greece or Poland. Similar conclusions are drawn by Bayrakdar and King [2] in their comparative study, in which they find similar patterns of discrimination, harassment and violence in Portugal, Germany and the United Kingdom. Nevertheless, having analysed the current state of affairs in Portugal, the “Eurobarometer on Discrimination 2019: The social acceptance of LGBTI people in the EU” [3] obtained data that were lower than the European mean in some questions referring to: the acceptance of same-sex relationships (EU mean: 72%; Portugal: 69%); the public display of affection between two men (EU mean: 63%; Portugal: 53%) and two women (EU mean: 68%; Portugal: 55%); being a work colleague of a gay, lesbian or bisexual person (EU mean: 82%; Portugal: 78%); acceptance of having a son or daughter in a same-sex relationship (EU mean: 67%; Portugal: 50%) or that school syllabuses include issues related to sexual orientation (media UE: 71%; Portugal: 68%). Lastly, the perception that a person can suffer discrimination is perceived as highly probable in Portugal (71%), in contrast to the European Union average of 53%.

Over the last few years, different studies have been undertaken in Portugal to analyse attitudes toward gay and lesbian people. Most of these studies [4,5] use Herek’s scale [6], although specific instruments have also been created for application in lusophone countries [7,8]. However, these instruments do not always comply with the recommendations for the study of these attitudes. On the one hand, the research recommends avoiding the use of scales that measure LG or LGBT as one [9]. The need for a separate analysis of attitudes toward gay men and lesbian women is based on the fact that each one of them has their roots in different types of prejudice, as well as different explanations of human sexuality and of sexual-affective relationships between men and women [10].

On the other hand, studies warn of the need to take subtle aspects into account in the discrimination against LGB people [4], which instruments such as Herek’s may not detect. Looking at the analysis of negative attitudes toward homosexuality, authors such as Morrison and Morrison [11] suggest using the term homonegativity. Homonegativity refers to negative affect, cognitions, and behaviors directed toward individuals who are perceived—correctly or incorrectly—to be gay or lesbian. In this regard, studies show that homonegativity can appear in two forms. First, they allude to traditional or old-fashioned homonegativity, based on traditional prejudice that reflects moral or religious objections against gay men and lesbian women. Second, they introduce the concept of modern homonegativity to refer to prejudice that is no longer based so much on moral objections but on ideas that “(1) gay men and lesbians are making illegitimate (or unnecessary) demands for changes in the status quo (e.g., spousal benefits); (2) discrimination against homosexual men and women is a thing of the past; and (3) gay men and lesbians exaggerate the importance of their sexual preference and, in so doing, prevent themselves from assimilating into mainstream culture” ([11], p. 18).Therefore, the analysis of modern homonegativity goes beyond attitudes of direct and explicit rejection and discrimination (e.g., insults, threats) to focus on more subtle and indirect attitudes [12,13,14]. This change in the nature of attitudes may be a response to the sociopolitical and legal advances some countries have gone through in recent years [1,15].

One consequence of the change in attitudes is the need to update the instruments used to measure them. Rye and Meany [14] carry out a comparison between three instruments that measure attitudes toward gay men and lesbians [11,16,17], and conclude that Morrison and Morrison’s scale “is more representative of how people express prejudice today” (p. 166). The adequate selection and application of scales designed to measure these types of subtle attitudes makes it possible to obtain data that are better adjusted to the real behaviour of people [11]. However, using scales whose items are directed at forms of explicit and direct rejection and discrimination can produce results that are not in keeping with the real attitudes of the population towards gay and lesbian people [11,18].

### The Modern Homonegativity Scale

The aim of the scale is to measure attitudes toward behaviours and policies that are friendly to gay men and lesbians (LG-friendly policies). Morrison and Morrison [11] studied modern homonegativity in Canada with a first version that had 50 items, which, following the validation study, was reduced to 24 items: 12 items to measure modern homonegativity toward gay men (MHS-G), and the same number for homonegativity toward lesbian women (MHS-L). Both scales had high levels of reliability in samples both of men (MHS-G = 0.91; MHS-L = 0.89) and women (MHS-G = 0.91; MHS-L = 0.85), and a unidimensional factor structure (MHS-G = 45% and MHS-L = 47% of the total variance) that differs from other, old-fashioned homonegativity scales (i.e., Attitudes Toward Lesbians and Gay Men Scale-Short-Form, ATLG-S, 16).

The MHS-G and MHS-L scales have been validated in the United States [18], and in European countries such as the UK [19] and Ireland [20]. Researchers such as Górska et al. [21] show the importance of applying these scales in countries with different characteristics to those where they have traditionally been applied. Research is also required on the differences in the predictors of homonegativity according to cultural or contextual characteristics [22]. More recently, the scales have been validated in Brazil [23]. This study concludes that the internal consistency, explained variance and goodness of fit of the indices of the single-factorial structures of HMS-G and HMS-L show that the Brazilian version is valid and reliable.

Therefore, taking into account (a) the legal, social and political changes that have taken place in Portugal in the last few decades, tied with (b) the scant use of instruments that fulfil the recommendations in the Portuguese context, and (c) the conclusions in the validation of the MHS in Brazil, this study aims to adapt and validate the MHS scale for Portugal.

Furthermore, the study of attitudes toward homosexuality has found considerable sociodemographic differences. Men tend to show more hostile attitudes toward LGB people than women [24,25,26,27]. Likewise, people with strong religious beliefs, people who are politically conservative, and/or with a low level of education, have more negative attitudes [26,28]. In contrast, being a gay or bisexual person is related to more positive attitudes toward sexual diversity [29], as occurs with those who have LGB friendships or have received specific training in this area [28].

This investigation also seeks to analyse the modern homonegativity of Portuguese university students toward homosexuality with regard to personal (gender identity and sexual orientation), sociodemographic and ideological (religion) variables. Therefore, following the aims of this study and the results of previous investigations, we postulated the following hypotheses:

**Hypothesis** **1:** *Men will show greater modern homonegativity than women toward gay and lesbian people.*

**Hypothesis** **2:** *The participating students who do not identify as heterosexual will show less modern homonegativity than those who do identify as heterosexual.*

**Hypothesis** **3:** *The participating students who profess a religion will show greater modern homonegativity than those who do not.*

## 2. Materials and Methods

### 2.1. Participants

In this study, we used non-probability convenience sampling. Six hundred and forty-one Portuguese college students between 18 and 27 years of age participated in this study (M_age_ = 21.23; SD = 1.88). Seventy-three percent (*n* = 468) of participants self-identified as female, 82.2% (*n* = 526) as heterosexual, and 51.2% (*n* = 328) stated they had a religion, mostly Catholic (93.8%). Most participants studied social science programmes, followed by biology/health science programmes. Table 1 displays the participants’ sociodemographic characteristics in greater detail.

### 2.2. Measurement Instruments

Sociodemographic Questionnaire. We asked participants about their age, their gender (female, male or other), their sexual orientation (heterosexual, bisexual, gay or lesbian, pansexual and other), their religion (yes or no), and their field of study.

Modern Homonegativity Scale. The Modern Homonegativity Scale (MHS) measures contemporary negative attitudes toward gay men and lesbian women and was originally developed in Canada by Morrison and Morrison [11]. We used the Portuguese version of the MHS which contains 22 items, two of which require reverse scoring (item 5 and item 15) and uses a 5-point Likert-type scale (1 = strongly disagree; 5 = strongly agree). Higher scores represent greater levels of modern homonegativity. Items 1 to 10 refer to negative attitudes toward gay men (examples of items include: “If gay men want to be treated like everyone else, then they need to stop making such a fuss about their sexuality/culture”, or “Gay men do not have all the rights they need”), and items 11 to 20 refer to attitudes toward lesbian women (examples of items include: “Lesbians who are ‘out of the closet’ should be admired for their courage”, or “If lesbians want to be treated like everyone else, then they need to stop making such a fuss about their sexuality/culture”). Items 21 and 22 measure overall negative attitudes and can be included when measuring attitudes toward gay men or lesbian women (examples of items are: “Celebrations such as “Gay Pride Day” are ridiculous because they assume that an individual’s sexual orientation should constitute a source of pride” and “The notion of universities providing students with undergraduate degrees in Gay and Lesbian Studies is ridiculous”). The translation and a retroversion of this set of items were corroborated independently by two specialists in Portuguese and English.

Attitudes Toward Lesbians and Gay Men Scale. We used the Portuguese version of the Attitudes Toward Lesbians and Gay Men Scale (ATLG) [8] originally developed by Herek [6]. The Portuguese validated scale consists of 24 items assessing three negative attitudinal factors: rejection of proximity, homosexuality pathologization and modern heterosexism; and one positive dimension: support. It uses a 5-point Likert-type scale (1 = strongly disagree; 5 = strongly agree). Since this is a validated scale and it was only used to assess convergent construct validity, we only used one overall measure of the ATLG scale, which is a direct mean of all items. Reliability analysis in the present study was very good (α = 0.87).

### 2.3. Procedures

Data collection was carried out online during the month of October 2021, through a website created for this purpose, which was disseminated through social networks targeting Portuguese college students. Using published email lists, we randomly contacted the coordinators of bachelor’s and master’s degrees at different Portuguese polytechnics and universities to make the request to their students to participate in this investigation. Anonymity, confidentiality, and informed consent were duly safeguarded. The information present in the Informed Consent was read and accepted by all participants and the data subsequently obtained were stored in encrypted databases, without reference to their IP’s. This study was approved by the Ethics Committee of the University of Granada (580/CEIH/2018).

### 2.4. Data Analyses

**Figure 1:**
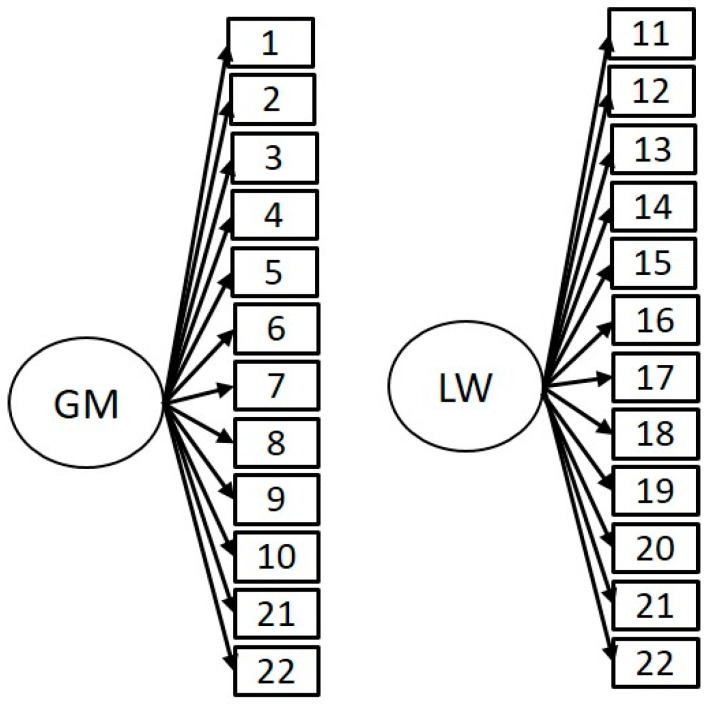
Proposed model of the MHS (GM = Gay Men; LW = Lesbian Women).

Data analyses were conducted separately for the male and female subscales of the MHS. Descriptive statistics, correlational analyses, exploratory factor analysis, and reliability analysis (Cronbach’s alpha coefficient) were calculated for all items of the MHS Scale using SPSS (v. 27, SPSS Inc., Chicago, IL, USA). The sensitivity of the items was assessed through Skewness (Sk) and Kurtosis (Ku) analysis. Absolute values of |Sk| and |Ku| higher than three and seven, respectively, were considered violations of the normality assumption. To conduct Confirmatory Factor Analysis (CFA), we used structural equation models using AMOS (v. 18, SPSS Inc. Chicago, IL, USA). The model’s fitness was evaluated through the following (Figure 1): χ^2^/df (Ratio Chi-square and Degrees of Freedom), CFI (Comparative Fit Index), PCFI (Parsimony CFI), RMSEA (Root Mean Square Error of Approximation). In order to analyse the modern homonegativity, we used inferential analysis (Student’s *t*). The effect size was analysed with Cohen’s d test. It was considered a small effect when d = 0.20, medium when d = 0.50, and large if d = 0.80. The statistical software used was SPSS (v. 27, SPSS Inc., Chicago, IL, USA).

## 3. Results

Taking into consideration that attitudes towards gay men and lesbian women can be seen as independent factors, we present the respective results separately. This was done having taken into consideration previous studies [11,18] that have studied instrument validation in this way.

Descriptive and distributional properties of the MHS for gay men and lesbian women (Table 2 and Table 3) show that the entire five-point Likert type scale was used for all items, with answers ranging from one to five. The distribution of the items had acceptable Skewness and Kurtosis values [30].

Regarding the exploratory factor analysis process, the Kaiser-Meyer-Olkin (KMO) coefficient was calculated. We obtained a score of 0.935 and a statistically significant Bartlett sphericity test χ^2^ (66) = 3091.237, *p* < 0.001 for the gay men scale; and a KMO of 0.933 and a statistically significant Bartlett’s sphericity test χ^2^ (66) = 3539.731, *p* < 0.001 for the lesbian women scale, suggesting that the data matrix is appropriate for performing an exploratory factor analysis. The analysis of the eigenvalues and the respective scree plot suggests the retention of a single factor, which explains 47.565% of the variance for the gay men scale, and 51.068% of the variance for the lesbian women scale.

The factorial weight of each item can be seen in Table 4. The unifactorial scale, comprising 12 items for each subscale, presents a Cronbach’s alpha value of 0.885 for the gay men scale, and 0.901 for the lesbian women scale, indicating excellent internal consistency.

Confirmatory factor analysis (CFA) was performed to determine the model goodness of fit with the variables and structure proposed by Morrison and Morrison [11]. The variables used were the variables for the MHS scales (gay men and lesbian women), which consisted of one single construct. The CFA test was conducted with the original scale characteristics. This model presented an acceptable fit to the data (see Table 5 for details). χ^2^/df, CFI, RMSEA and PCFI presented a good fit to the data.

To assess the convergent validity of the gay men and lesbian women MHS scales, Pearson’s correlations were calculated between the scales ‘MHS-gay men’, ‘MHS- lesbian women’, and ‘Overall ATLG’ (Table 6). All correlations were found to be statistically significant (*p* < 0.01), positive and strong, indicating convergent validity.

We also analysed modern homonegativity in relation to the gender, age, sexual orientation, and religion of the students.

Regarding gender, the male students (Gays: M = 2.49; DT = 0.79; Lesbians: M = 2.44; DT = 0.81) showed significantly greater modern homonegativity toward gay men [t (225.46) = −8.93; *p* < 0.001; Cohen’s d = 0.95] and lesbian women [t (223.91) = −8.78; *p* < 0.001; Cohen’s d = 0.94] than the female students (Gays: M = 1.90; DT = 0.56; Lesbians: M = 1.84; DT = 0.57). These differences showed a large effect size.

According to sexual orientation or choice (heterosexual vs. non-heterosexual), the results revealed statistically significant differences, with a medium effect size, in the showing of modern homonegativity toward gay men [t (218.89) = 8.03; *p* < 0.001; Cohen’s d = 0.67] and lesbian women [t (239.75) = 9.35; *p* < 0.001; Cohen’s d = 0.74]. The heterosexual participants revealed greater modern homonegativity toward both groups (Gays: M = 2.12; DT = 0.69; Lesbians: M = 2.07; DT = 0.70) than the rest (Gays: M = 1.68; DT = 0.49; Lesbians: M = 1.58; DT = 0.46).

Lastly, we analysed the differences in the showing of modern homonegativity between religious believers and non-believers. Those participants who professed a religion expressed greater modern homonegativity toward homosexual people than those who stated they had no religion. However, these differences were not significant.

## 4. Discussion

This study had two aims. First, we aimed to adapt and validate the Modern Homonegativity Scale [11] for use in Portugal. Second, we attempted to analyse the modern homonegativity of Portuguese university students with regard to certain sociodemographic, personal, and ideological variables.

For the first aim, the Exploratory Factor Analysis produced results for the KMO and Bartlett’s sphericity that are similar to other validation studies of this instrument [23]. This indicates the existence of one sole factor of modern homonegativity in both subscales, obtaining a percentage of explained variance around 50% and an excellent internal consistency [11,18,23]. The analysis of some psychometric properties of the items reflect a good response distribution, and a factor loading higher than 0.30 [11,31]. Nevertheless, the items that have reverse scoring (item 5 and item 15) present a low factor loading. This lower factor loading could be due to the method effect that is found in scales with negatively worded or reversed items, and which has been studied in self-report measures of personality variables [32]. In research, it was initially recommended to use reversed items to prevent acquiescence bias and other response bias. However, more recent research [33] has shown that these reversed items require greater verbal comprehension. We therefore suggest that, in future studies, the original phrasing of the items be improved. 

The results concur with what has been found in previous studies [11,14,18,23]. The single-factor construct used for the CFA presents acceptable fit indices for both scales, as per the suggested values for an acceptable fit (CFI ≥ 0.90; RMSEA ≤ 0.08) proposed by Morrison, Morrison and Franklin [18]. Furthermore, various recent validation studies show similar fit values (23, in Brazil; 21, in Poland). The MHS is thus considered a scale with good construct validity.

Regarding the convergent validity, the results show that modern homonegativity has a strong and positive correlation with a traditional manifestation of attitudes toward gay men and lesbians (ATLG) [14,34]. Examining racism and modern sexism, Morrison and Morrison [11] determine that both the modern and traditional manifestations of homonegativity are interrelated, despite being conceptually different. This could be due to the fact that both modern and traditional prejudices entail a rejection of minority sexual groups [20]. Therefore, it appears that those people who show greater modern homonegativity also exhibit a greater traditional manifestation of negative attitudes toward gay men and lesbian women. Hence the external validation of this scale is in line with previous studies [14].

With regard to the second objective, as expected (Hypothesis 1) the men show higher modern homonegativity toward gay men and lesbian women. These results are in agreement with other studies [24,25,26,27,35], which establish that men are compelled to comply with a model of hegemonic masculinity that rejects homosexuality, above all male homosexuality, as well as non-traditional manifestations of gender. 

Likewise, the results obtained in terms of sexual orientation also confirm our hypothesis (Hypothesis 2). Those people who identify as heterosexual reveal greater modern homonegativity toward gay and lesbian people [29]. This fact could be due to the idea that homosexuality challenges heterosexual relations and practices, and identity and gender roles, which are normalized through heteronormativity [25].

However, religion is not related to the expression of modern homonegativity toward gay men and lesbians. In contrast to what we anticipated (Hypothesis 3), there are no significant differences between those who professed a religion and those who did not. This finding contradicts other studies [26,36] that indicate the influence of religiousness on homophobic attitudes toward gay and lesbian people. Nevertheless, these findings could be explained by the relative influence of religion in Portuguese society. D’Amore et al. [1] conclude that religion is less conservative in some European countries than in others. They also show that the degree of religiousness has an influence on attitudes, with greater religious commitment being related to more negative attitudes. Sherkat, De Vries & Creek [37] also had similar findings.

The findings of this study aim to be an advance in the research of this field. In this regard, we have considered different implications in the university setting. It has been noted that the manifestation of homonegativity in higher education has evolved toward modern forms [11]. This study has evolved in response to the need to use instruments that take this into account and are able to provide a better adapted approach to the LGBT reality in university classrooms [11,14]. Our results therefore make it possible to explore the attitudes of university students toward homosexuality. In line with other studies [38,39], we highlight the need to understand this aspect in order to design better adapted and adjusted curricular programmes with the purpose of offering an inclusive university education that is respectful of homosexual students. This investigation is focused on analysing attitudes toward the gay and lesbian population. It is also relevant for the design and validation of specific instruments in the Portuguese context that analyse attitudes toward other groups, such as bisexual and transgender people.

In addition, the acquisition of knowledge about LGBT matters is related to greater positive attitudes toward these students [40,41]. Yet various studies [42] have pointed out the scant inclusion of content on sexual-affective diversity in university syllabuses. By way of response, this study shows that universities should consider the possibility of offering training specifically on these issues. Lastly, these results might have implications for university guidance and counselling services. It would be appropriate to work on strategies aimed at promoting an inclusive university environment, such as producing inclusive language guides or holding awareness campaigns, among other strategies [43].

In terms of this study’s limitations, we should point out both the size and distribution of the sample, and the online data collection. The study could be improved by increasing the number of male participants and of other, non-heterosexual orientations. This having been said, the use of offline and online questionnaires does not affect the quality of the results [44]. Another limitation concerns the variables used in the study. Previous studies [34,35] highlight the influence that different sociodemographic and personal variables (such as age or LGBT friendships), and ideological variables (e.g., political inclinations or belief about the cause of homosexuality), have on a greater or lesser display of homonegativity. Similarly, the training received on this topic can have a positive effect on attitudes toward LGB people. For future studies, we consider it necessary to take these aspects into account when analysing homonegativity in order to obtain better adjusted and more profound results.

## 5. Conclusions

In general terms, the results suggest that the Portuguese version of the MHS is valid and reliable for evaluating modern homonegativity in Portugal. Following the findings of similar studies, we recommend the use of this instrument, since it examines the contemporary forms of homonegativity present in university students. In this regard, it has shown how certain personal and sociodemographic variables influence modern homonegativity. We need to continue studying the psychometric properties of this scale both to delve into the effect of the inverted items and the dimensionality of both scales, as the conception of homonegativity can alter with social change.

## Figures and Tables

**Table 1:** Sociodemographic characteristics (*n* = 641; M_age_ = 21.23; SD = 1.88).

		*n*	%
Gender	Female	468	73%
	Male	165	25.7%
	Other	8	1.3%
Sexual orientation	Heterosexual	526	82.2%
	Bisexual	57	8.9%
	Gay/Lesbian	35	5.5%
	Pansexual	15	2.3%
	Other	7	1.1%
Religion	Yes	328	51.2%
	No	313	48.8%
Field of study	Social Sciences and Education	363	56.6%
	Arts and Humanities	74	11.6%
	Engineering and Data Sciences	100	15.6%
	Health/Biological sciences	104	16.2%

**Table 2:** Descriptive and Distributional Properties of MHS—Gay Men Items.

Item	Mean	SD	Min	Max	Skewness	Kurtosis
1	2.3089	1.10598	1.00	5.00	1.008	0.413
2	2.1956	1.12296	1.00	5.00	0.569	−0.719
3	1.7473	0.92960	1.00	5.00	1.143	0.736
4	2.5438	1.07378	1.00	5.00	0.225	−0.726
5	2.5869	1.01318	1.00	5.00	0.288	−0.321
6	2.0375	1.02634	1.00	5.00	0.761	−0.261
7	1.5994	0.76915	1.00	5.00	1.385	2.240
8	2.2355	1.03301	1.00	5.00	0.530	−0.280
9	1.7402	0.90008	1.00	5.00	1.220	1.122
10	2.0329	1.14718	1.00	5.00	0.931	−0.086
21	1.9249	1.10314	1.00	5.00	1.020	0.148
22	2.0000	1.09545	1.00	5.00	1.062	0.418

**Table 3:** Descriptive and Distributional Properties of MHS—Lesbian Women Items.

Item	Mean	SD	Min	Max	Skewness	Kurtosis
11	1.7840	0.91161	1.00	5.00	1.075	0.738
12	2.3039	1.01125	1.00	5.00	0.392	−0.555
13	1.9249	0.98452	1.00	5.00	0.912	0.158
14	2.0801	1.07203	1.00	5.00	0.685	−0.525
15	2.6604	1.06382	1.00	5.00	0.309	−0.371
16	2.2166	1.05516	1.00	5.00	0.550	−0.337
17	1.9906	1.10369	1.00	5.00	0.925	−0.091
18	1.7555	0.92400	1.00	5.00	1.270	1.314
19	1.6066	0.77869	1.00	5.00	1.273	1.450
20	2.0207	0.95072	1.00	5.00	0.897	0.623
21	1.9249	1.10314	1.00	5.00	1.020	0.148
22	2.0000	1.09545	1.00	5.00	1.062	0.418

**Table 4:** Results of the Exploratory Factor Analysis: Factor loadings, Cronbach’s alphas and % of Variance of the unifactorial analysis of the MHS.

	Gay Men		Lesbian Women	
Factor loadings	Item 1	0.547	Item 11	0.822
	Item 2	0.447	Item 12	0.722
	Item 3	0.777	Item 13	0.740
	Item 4	0.735	Item 14	0.506
	Item 5	0.264	Item 15	0.282
	Item 6	0.684	Item 16	0.700
	Item 7	0.792	Item 17	0.836
	Item 8	0.705	Item 18	0.810
	Item 9	0.780	Item 19	0.825
	Item 10	0.848	Item 20	0.699
	Item 21	0.791	Item 21	0.778
	Item 22	0.670	Item 22	0.657
α		0.885		0.901
% of variance		47.565		51.068

**Table 5:** Goodness of fit indices for CFA of the MHS.

	MHS Gay Men	MHS Lesbian Women
χ^2^/df	228.522/54	249.202/54
CFI	0.960	0.955
RMSEA	0.064	0.077
PCFI	0.665	0.634

**Table 6:** Convergent Construct Validity.

	1	2	3
1-MHS–Gay Men	-		
2-MHS–Lesbian Women	0.965 **	-	
3-Overall ATLG	0.665 **	0.684 **	-

** *p* < 0.001.

## Data Availability

The data presented in this study are available upon request.
